# Horizontal gene transfer-mediated bacterial strain variation affects host fitness in *Drosophila*

**DOI:** 10.1186/s12915-021-01124-y

**Published:** 2021-09-27

**Authors:** Yun Wang, Franz Baumdicker, Paul Schweiger, Sven Kuenzel, Fabian Staubach

**Affiliations:** 1 Department of Evolutionary Biology and Ecology, Institute of Biology I, Albert Ludwigs University Freiburg, Freiburg, Germany; 2 Cluster of Excellence ‘Controlling Microbes to Fight Infections’, University of Tübingen, Tübingen, Germany; 3 Department of Mathematical Stochastics, University of Freiburg, Freiburg, Germany; 4 Microbiology Department, University of Wisconsin – La Crosse, La Crosse, WI USA; 5 Max Planck Institute for Evolutionary Biology, Ploen, Germany

**Keywords:** Drosophila, Microbiome, GWAS, Horizontal gene transfer, Lateral gene transfer

## Abstract

**Background:**

How microbes affect host fitness and environmental adaptation has become a fundamental research question in evolutionary biology. To better understand the role of microbial genomic variation for host fitness, we tested for associations of bacterial genomic variation and *Drosophila melanogaster* offspring number in a microbial Genome Wide Association Study (GWAS).

**Results:**

We performed a microbial GWAS, leveraging strain variation in the genus *Gluconobacter*, a genus of bacteria that are commonly associated with *Drosophila* under natural conditions. We pinpoint the thiamine biosynthesis pathway (TBP) as contributing to differences in fitness conferred to the fly host. While an effect of thiamine on fly development has been described, we show that strain variation in TBP between bacterial isolates from wild-caught *D. melanogaster* contributes to variation in offspring production by the host. By tracing the evolutionary history of TBP genes in *Gluconobacter*, we find that TBP genes were most likely lost and reacquired by horizontal gene transfer (HGT).

**Conclusion:**

Our study emphasizes the importance of strain variation and highlights that HGT can add to microbiome flexibility and potentially to host adaptation.

**Supplementary Information:**

The online version contains supplementary material available at 10.1186/s12915-021-01124-y.

## Background

Microbes are important drivers of host phenotype and evolution [1]. Benefits derived from microorganisms can facilitate the occupation of new ecological niches [2–5] and microbial effects on host phenotypes and fitness can spur adaptive processes [6–14]. Changes in the effects of microbes on host fitness can alter interactions along the parasitism mutualism continuum [6, 15–18], thus affecting the evolutionary trajectories of the partners. The importance of microbes in evolution and health of higher organisms has sparked a search for the molecular underpinnings of how microbes affect host phenotype.

In this search, microbial Genome Wide Association Studies (GWAS) are an important tool [19–23]. The principle behind a microbial GWAS is to establish a link between traits and genetic variation of microbes by the means of GWAS. By testing for association between host traits and microbial genomic variation, Chaston et al. [24] introduced a particularly helpful approach to unravel how microbes affect host phenotypes [22, 24]. The authors measured host phenotypes, here from *Drosophila melanogaster* that were mono-associated with several microbial isolates. Differences in host phenotype were then associated with the presence and absence of genes in the microbial isolates. By applying this approach, it was found that genes that play a role in glucose oxidation in bacteria affect *D. melanogaster* triglyceride levels [24] and that bacterial methionine and B vitamins are important for starvation resistance [25] as well as life span [26].

For targeting host phenotypes with microbial GWAS, model systems that allow the generation of axenic hosts that can successively be associated with individual microbial isolates are particularly useful [24]. One such model system is *D. melanogaster* and its bacterial microbiome. Techniques for the generation of gnotobiotic flies are readily available and standardized measurements of phenotypes exist. Microbe-affected host phenotypes include the life history traits such as development time, fecundity, and life span as well as size of the adults [14, 26–33]. These traits are components of fitness, emphasizing the potential importance of microbes in host evolution and adaptation [14, 32]. Microbes often affect fitness-related traits by provisioning nutrients. These nutrients include vitamins, amino acids, lipids, and trace elements [24, 34–39]. Nutrient provisioning is a recurring theme in metazoan–microbe interactions that are adaptive for the host [40–42].

The acquisition of nutrients from microbes need not rely on microbes that live inside the host. Instead, nutrients can also be acquired by harvesting or preying upon microbes that live outside the fly and subsequent digestion [35, 43–45]. Furthermore, bacteria have been identified that affect *D. melanogaster* phenotypes by increasing the ability for nutrient uptake [46] or metabolizing components of the food substrate, and thus modulating its nutrient content [24]. Interestingly, the metabolic potential to produce nutrients that affect fly fitness differs between closely related microbes and so do the effects on fly phenotype and fitness [26, 29, 32, 47–51]. These findings contribute to the notion that microbial variation at taxonomically low levels is not only important for human [15], mouse [52], and plant [18] hosts, but also for *Drosophila* [53].

Because variation between closely related bacteria is important for the interaction of the host and its microbiome, it is also important to consider closely related microbes in studies that aim at elucidating the molecular underpinnings of host–microbe interaction. At the same time, limiting a GWAS to the pan-genome of closely related microbes might offer particular power to the approach: limitation to a narrower range of genes that vary in their presence-absence patterns in a similar genomic background, microbial genes that affect the host can be more precisely pinpointed. For studies that are aimed at better understanding host–microbe interaction in an evolutionary context, it is also important to consider microbes that are associated with the host under natural conditions and if possible, to measure evolutionary relevant host phenotypes in a natural or near natural environment. Finally, tracing the evolutionary changes of the genomic elements that affect host fitness can help us to gain deeper insights into how host–microbe interaction evolves.

We aimed our study at better understanding whether and how fly fitness is affected by its natural microbiome by a microbial GWAS. In order to increase the resolution of the approach and consider variation at low taxonomic levels, we concentrated on variation within a taxonomically restricted group of bacteria. Therefore, we focused our study on *Gluconobacter,* a bacterial genus that is commonly associated with *D. melanogaster* under natural conditions [54–57]. We assessed offspring number per female fly as a fitness component on grape juice-based fly food as a near natural food source. In order to better understand how microbial effects on host fitness evolve, we traced the evolutionary events that lead to changes in bacteria-mediated host fitness.

## Results

**Fig. 1:**
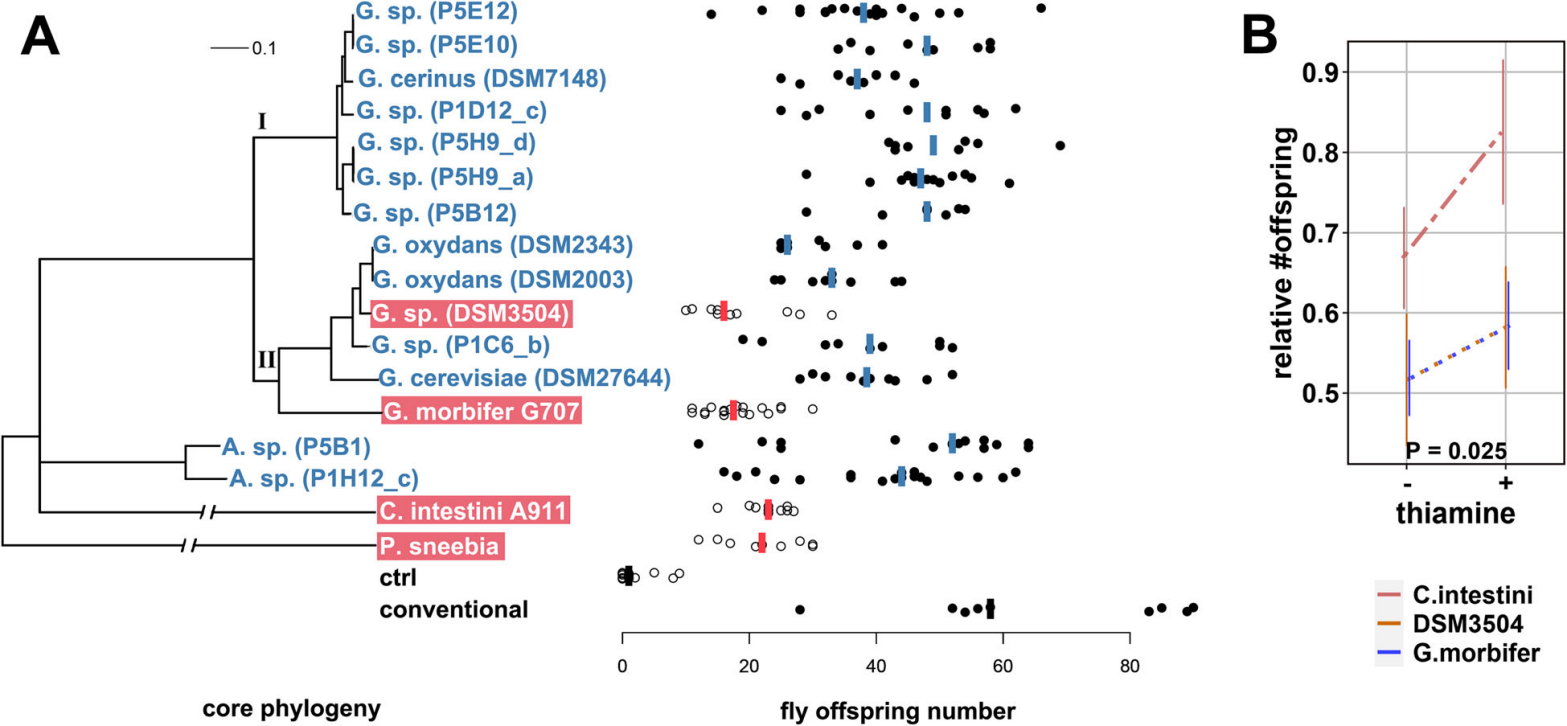
**A** Left: Bacterial tree based on 134 single-copy orthologs. Bootstrap support is 100% for all nodes. Leaf labels of bacteria that do not carry a complete thiamine biosynthesis pathway are on red background. Right: Number of offspring produced by mono-associated CantonS females; vertical bars: median; ctrl: axenic flies; conventional: flies reinfected with lab microbiota. **B** Thiamine treatment (1 μg/ml added to the food) increased the relative number of offspring for the strains that do not possess a complete thiamine biosynthesis pathway (TBP−) compared to strains that possess the complete pathway (TBP+). Relative offspring number was determined by dividing the number of offspring for each TBP− strain by average offspring number of the TBP+ strains. A value of one would mean equal offspring number between TBP+ and TBP− strains. *P*-value was determined with a linear mixed effects model. Error bars indicate standard error of the mean (Additional file 4: Script S1)

We performed a microbial GWAS for the number of offspring produced by females that were mono-associated with 17 bacterial isolates from genera that co-occur with *Drosophila melanogaster* in its natural environment. *Gluconobacter* was represented by 13 isolates. Two additional isolates were from the genus *Acetobacter*. Species from this genus can benefit *Drosophila* development [28]. One isolate was *Commensalibacter intestini* that might have a probiotic function in *D. melanogaster* [58] and is enriched in flies over substrate in wild-caught flies [57]. As an outgroup and to get a baseline for the fitness effect of an ingested pathogen, we added *Providencia sneebia* that is highly pathogenic when entering the hemolyph [59]. All bacterial genomes analyzed were >99% complete with the exception of *P. sneebia* (>96%, Additional file 1: Table S1). The mean number of offspring varied significantly between flies mono-associated with different isolates (*P* = 4.2 × 10^−15^, Kruskal-Wallis test, Fig. 1A) up to a 2.8-fold difference between *Gluconobacter morbifer* and *Gluconobacter sp.* P5H9_d. Differences between bacterial strains were also a significant covariate of offspring number when we accounted for bacterial loads per fly (*P* = 1.4 × 10^−4^, linear model). Furthermore, bacterial load alone was not significantly associated with fly offspring number (*P* = 0.11, linear model), suggesting that not only bacterial biomass affects fly fitness. Presence-absence patterns of 11,269 genes were tested for association with the number of offspring that mono-associated females produced using the PA method [60]. Associations were confirmed by permutation tests and TreeWAS [21] (Table 1, Additional file 2 and 3: Figure S1, Table S2). The six highest PA scores depended strongly on presence-absence patterns between the closely related strains P1C6_b, DSM2003, DSM2343, and DSM3504 (mean ANI = 95.5%) in the branch that includes *G. morbifer* (Additional file 2: Figure S1 and accompanying text).

**Table 1:** List of the ten genes that were most strongly associated with offspring number according to PA-association scores. All associations were confirmed by at least one of three methods from treeWAS [21]

Gene name	Annotation	PA score	treeWAS terminal	treeWAS simultaneous	treeWAS subsequent
			***p***-value	***p***-value	***p***-value
	Transposase	4.62	0.000009	0.013	0.003
ThiO	Putative thiamine biosynthesis oxidoreductase	4.34	0.0002	0.003	0.02
ThiG	Thiazole synthase	4.34	0.0002	0.003	0.02
ThiS	Thiamine biosynthesis protein	4.34	0.0002	0.003	0.02
ThiC	Thiamine biosynthesis protein	4.34	0.0002	0.003	0.02
ThiD	Phosphomethylpyrimidine kinase	4.34	0.0002	0.003	0.02
	Ferric iron siderophore receptor	4.21	0.000009	0.11	0.001
	Oxidoreductase	4.21	0.000009	0.11	0.001
	LysR family transcriptional regulator	4.21	0.000009	0.11	0.001
	Methyltransferase domain protein	4.06	0.0003	0.009	0.89

### The bacterial thiamine biosynthesis pathway is associated with increased offspring number

Five of the six bacterial genes that were most strongly associated with offspring number were part of the thiamine biosynthesis pathway (TBP, Table 1). Females reared on bacteria carrying a complete TBP (TBP+) produced more offspring (*P* = 0.0038, Mann-Whitney Test on strain medians, *n* = 17, Fig. 1A), suggesting that bacterial thiamine production might increase the number of offspring.

Because high numbers of *Drosophila* offspring on a confined resource like a *Drosophila* vial can lead to crowding effects, including smaller adults and reduced individual fitness, we weighed the adult flies at the end of the experiment. Weight did not differ significantly between the offspring of females reared on TBP+ and TBP− strains (*P =* 0.55, Mann-Whitney Test on strain medians, *n* = 17, Additional file 2: Figure S2), providing no evidence for larval crowding or reduced adult size. Significance of all *p*-values was confirmed in a linear model framework that accounts for bacterial load and also in a phylogenetic ANOVA (Additional file 4: Script S1).

We hypothesized that if bacterial thiamine increased the offspring number of females reared on TBP+ strains, supplementing the diet of flies reared on TBP− strains with thiamine would increase offspring number when compared to TBP+ strains. To test this, we supplemented the diet of females that were mono-associated with TBP− strains (*G. sp.* DSM3504, *G. morbifer* G707, *C. intestini* A911) with thiamine and applied the same assay for offspring number as for the initial GWAS. Supplementing the diet of flies reared on closely related TBP+ strains served as control (*G. oxydans* DSM2343, *G. oxydans* DSM2003, *G. sp.* P1C6_b, *G. cerevisiae* DSM27644). In order to account for variation between experiments, we calculated the relative offspring number between TBP− and TBP+ strains in the initial unsupplemented experiment (“thiamine treatment −”, Fig. 1B) and the supplementation experiment (“thiamine treatment +”, Fig. 1B). Indeed, the relative offspring number on TBP− strains increased with thiamine supplementation (*P* = 0.025, linear mixed effects model, Fig. 1B), supporting a role of thiamine production in the number of offspring that flies produced. We found no evidence that the addition of thiamine increased bacterial loads of TBP− strains (*P* = 0.85, generalized linear model), suggesting that the increase in offspring number is not due to an increase in bacterial biomass alone.

### Thiamine biosynthesis genes were most likely lost and reacquired by horizontal gene transfer as an operon on the branch that includes *G. morbifer*

**Fig. 2:**
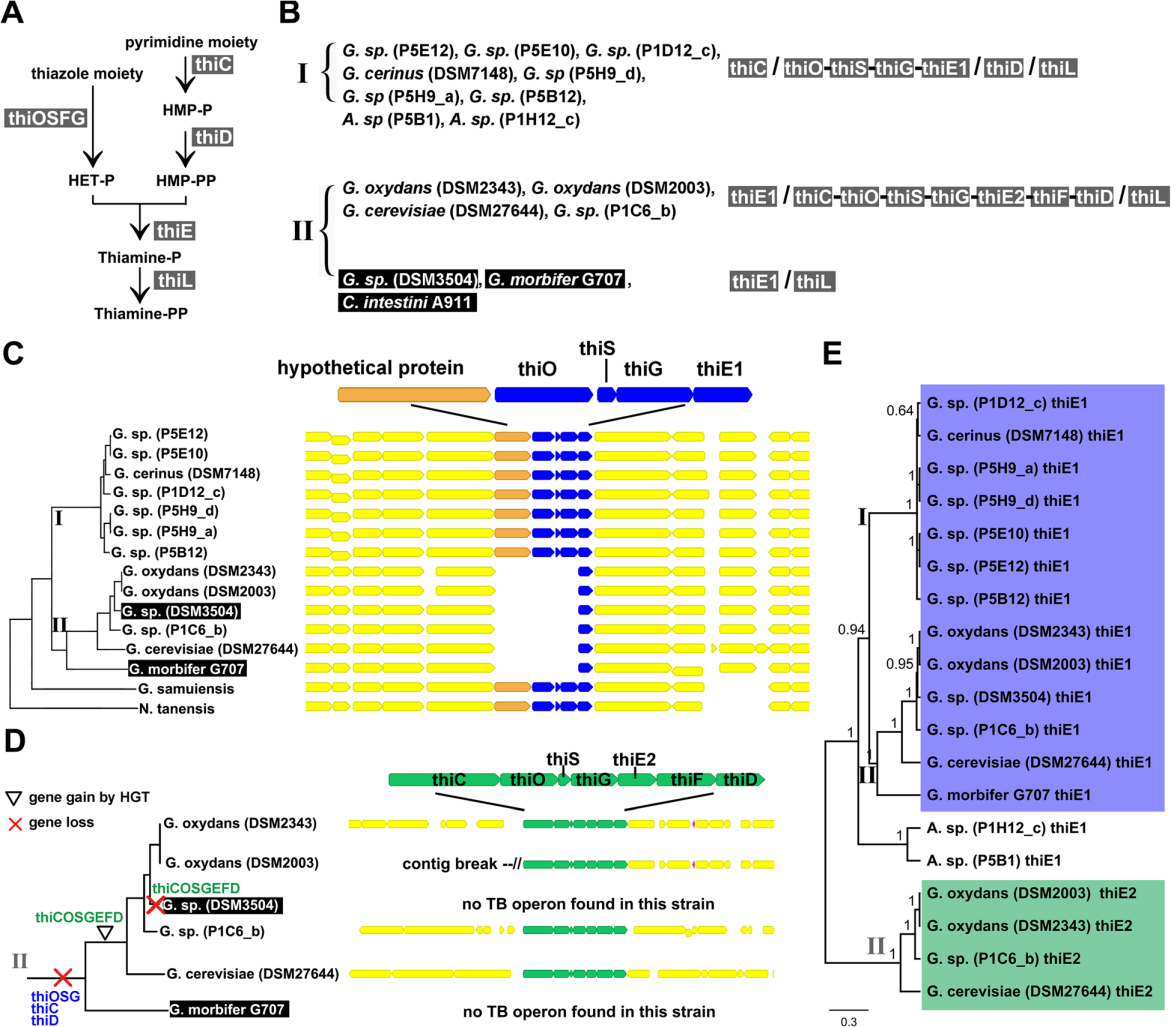
**A** The thiamine biosynthesis pathway in acetic acid bacteria. **B** Overview of thiamine biosynthesis genes in the analyzed bacteria. Note that the function of thiF that appears to be missing in the strains of the upper row can be replaced by the function of the homolog MoeB (Rodionov et al., 2002) that we found in all strains analyzed. Genes forming one operon are separated by a hyphen. Genes from different loci are separated by slashes. **C** Synteny of the flanking regions of thiamine biosynthesis genes in *Gluconobacter* and *Acetobacter*. thiOSG are missing on the *G. morbifer* branch (II) at this locus. Thiamine biosynthesis genes are in blue. The hypothetical protein is of unknown function. **D** Right: The complete pathway to synthesize Thiamine-P (green) forms an operon on the G. morbifer branch (branch II); left: the phylogeny depicts the inferred evolutionary scenario on branch II. **E** Phylogeny of thiE. *G. oxydans* DSM2343, *G. oxydans* DSM2003, *G. sp.* P1C6_b, and *G. cerevisiae* DSM27644 have two copies of thiE, thiE1 (blue) and thiE2 (green). The phylogeny of thiE1 (blue background) is congruent with the core genome phylogeny. ThiE2 (green background) forms a distinct clade that is more distant than thiE from Acetobacter, indicating HGT from a distant clade. Node labels represent posterior probabilities as assessed by MrBayes v 3.2.6 [62]

In order to better understand the evolutionary history of the TBP (Fig. 2A) in *Gluconobacter*, we analyzed the synteny of the underlying loci in a phylogenetic framework. The strains in the upper two panels of Fig. 2B possess all genes required for thiamine biosynthesis. A closer inspection of TBP genes on the *G. morbifer* branch (II in Fig. 2C) revealed that two strains are TBP−, while the four other strains are TBP+. Inspection of the TBP gene loci revealed that all strains on branch II are missing the operon-like structure thiOSG (Fig. 2C) at the locus that is syntenic with branch I. The same pattern was found for thiC and thiD (Additional file 2: Figure S3). thiOSG (Fig. 2C), thiC, and thiD (Additional file 2: Figure S4) are present in the closely related bacteria *Gluconobacter samuiensis* and *Neokomagateaa tanensis* at syntenic loci, suggesting deletion on branch II. The strains with an intact operon on branch II carried a TBP operon at loci not syntenic with the locus shown in Fig. 2C as evident from different flanking genes (Fig. 2D, Additional file 2: Figure S5), suggesting insertion.

Analyzing the sequences of the inserted genes in a phylogenetic framework, we found that the inserted genes form a distant clade. For example, thiE1, the copy that remained at the locus shown in Fig. 2C, followed the phylogeny based on the core genome, while the potentially newly acquired copy thiE2 that is part of the operon thiCOSGEFD formed a distant clade (Fig. 2E), supporting HGT. Within this clade, the phylogeny of thiE2 is again congruent with the core genome phylogeny, consistent with a single reacquisition event of thiCOSGEFD. The same phylogenetic patterns were found for the other TBP genes that were shared across branches (thiCOSGD, Additional file 2: Figure S6), further supporting a single HGT of thiCOSGEFD to the *G. morbifer* branch. Because TBP genes can occur on plasmids [61], we blast searched the plasmids of the strains for which the plasmids were resolved for TBP genes, finding no evidence for TBP genes (data not shown). In order to identify a potential donor of the operon, we blast searched the sequence of the entire operon against the ncbi non-redundant nucleotide database (nr). The best matching non-*Gluconobacter* sequences were from Rhodobacteraceae, a phylogenetically distant bacterial family (Additional file 5: Table S3). A closer inspection of the non-*Gluconobacter* blast hits with the highest scores (query coverage 79–82%, ~73% identity, Additional file 5: Table S3) revealed that gene order within the operon, but not synteny of flanking genes, was conserved (Additional file 2: Figure S7). Despite a modest difference in GC-content between the potential donors (~66% vs 62% in *Gluconobacter oxydans* DSM2343), the GC-content of the putatively inserted operon did not differ from that of the genomic background (Additional file 2: Figure S8), providing no evidence for a recent acquisition from any of the top 3 blast hits. Furthermore, the best non-*Gluconobacter* blast hits were in marine bacteria. Taken together, this implies that the true donors remain enigmatic. A single reacquisition event of the essential TBP genes in the past, close to the base of clade II, as suggested by the concordance of the inserted operon with the core gene phylogeny, implies that the TBP− strain DSM3504 lost the operon again in an independent event, as depicted in Fig. 2D (left).

## Discussion

### Microbial GWAS for host traits can benefit from strain level variation

We applied a microbial GWAS approach that associates bacterial genes with host phenotype focusing on the genus *Gluconobacter*. Microbial GWAS approaches can be particularly powerful, when pan-genomic variation of closely related bacterial strains can be leveraged, as has been shown for, e.g., virulence genes [63]. We showed that genetic variation below the species level between the strains P1C6_b, DSM2003, DSM2343, and DSM3504 (mean ANI = 95.5%) empowered us to pinpoint the TBP (Additional file 2: Figure S1). Variation between bacteria that have ANI > 95% is considered strain level variation [64]. The only gene that had a higher association score for offspring number than the TBP genes was a transposase. Transposases more frequently produce rare presence-absence patterns because they are mobile and not linked as strongly to the rest of the genome as are non-mobile genetic elements. Therefore, we suspect that the high association score is an artifact of its mobility although we cannot exclude an effect of the transposon on the number of fly offspring. From the other genes with significant associations with offspring number presented in Table 1, the oxidoreductase, LysR family transcriptional regulator, and the methyltransferase domain protein, a plausible link to fly offpsring number is more difficult to test. Nonetheless, these genes might also affect fly offspring number. The ferric iron siderophore receptor is located close to the inserted thiamine operon in P1C6_b and DSM27644 which also possesses a gene with the same annotation at that locus, as is apparent from Additional file 2: Figure S5. While it seems possible that this gene contributes to fly fitness, it must be considered that DSM3504 that lacks TBP genes and confers relatively low fitness to the host also carries a ferric iron siderophore receptor that is orthologous to that shown in Additional file 2: Figure S5 in DSM27644. Given this and the demonstrated fitness effects of the thiamine supplement (Fig. 1B), we must assume that this gene received a high association score mainly due to linkage to the TBP genes.

### Variation between closely related microbes is important for host phenotypes

We observed significant variation of phenotypes between flies that were associated with closely related microbial strains. This supports the notion that strain level variation is important to consider when studying host–microbe interaction in animals, humans, and plants alike (e.g. [15, 18, 52, 65, 66]. In particular, in *D. melanogaster*, evidence for the importance of variation between closely related bacteria is accumulating for life history of the host [26, 29, 47–51, 67]. Unawareness of strain level variation in bacterial effects on the host might have led to perceived inconsistency between studies [53]. Furthermore, our study provides an example of the limits of 16S rRNA sequencing in functional inference: The 16S rRNA gene sequences, as assessed by full length Sanger sequencing of strains P1C6_b (TBP+) and DSM3504 (TBP−), were identical (Additional file 2: Figure S9).

### The number of offspring as a component of fitness

As a fitness component, we assessed the number of adult offspring produced after 16 days. As such, our assay captures developmental rates and fecundity on a time scale that we consider highly relevant for the reproductive success of an organism that is adapted to an ephemeral resource, rotting fruit [68]. The effect of thiamine on offspring number that we describe is consistent with previously described effects of bacterial thiamine on *D. melanogaster* development and survival to adulthood [37]. Yet, a limitation of our study is that other components of life time reproductive success, and thus fitness, were not directly assessed, in particular egg laying and longevity. However, Sannino et al. [37] also showed that bacterial thiamine neither affects egg laying nor longevity in *D. melanogaster.* This suggests that the effect of thiamine on the number of offspring that we observed is directly related to lifetime reproductive output, and thus fly fitness.

### The loss and regain of the TBP by HGT in the context of the evolution of host–microbe interaction

Offspring number was strongly associated with genes from the TBP. The acquisition of B vitamins like thiamine (B1) is a typical benefit that insects receive from microbes [25, 69, 70] and falls into the greater context of nutrient provisioning by microbes, which is a recurring theme in the evolution of host–microbe interaction [40, 41].

By tracing the genes of the TBP across genomes and the phylogeny, we found that the pathway to produce Thiamine-P was regained most likely via HGT (Fig. 2D). As such, our study exemplifies that individual events of HGT into a host-associated microbe can alter host fitness outcomes. Other studies that show an effect on host fitness via HGT to a host-associated microbe involve defensive compounds produced by microbial symbionts in plants [71] and animals [17, 72]. In our study, the increase in host fitness with the reacquisition of the TBP is most likely mediated via nutritional benefits. Only a few similar cases have been described so far. The most prominent may be the acquisition of vitamin B7 (biotin) and vitamin B2 (riboflavin) synthesis by planthopper-associated *Wolbachia* [73]. Similarly, bed bug-associated *Wolbachia* [74] and cat flea-associated *Wolbachia* seem to have gained the ability to produce biotin via HGT [75]. In ticks, *pabA* (and possibly *pabB*) required for the synthesis of folic acid was acquired by a *Coxiella*-like symbiont through horizontal gene transfer (HGT) from an Alphaproteobacterium [76] and is thought to affect tick fitness. A *Rickettsia* endosymbiont of deer ticks has acquired the genes necessary for the synthesis of biotin on a plasmid [77]. In this study, as in ours, a complete operon has been transferred.

A difference between our and these previous studies is that although *Gluconobacter* is frequently associated with *D. melanogaster* under natural conditions [54–57], it is neither an obligate symbiont nor is it restricted to the fly gut. While it is interesting that the natural fly isolates of *Gluconobacter* are TBP+, there is currently no evidence that the fly host significantly affects *Gluconobacter* evolution given its occurrence in the environment and the opportunity for horizontal transfer of the bacterium between hosts. Further, taking into account evidence that the abundance of mobile metabolic genes is governed by selection [78, 79], we must assume that loss and gain of the TBP must first of all benefit the bacterium to persist in the bacterial genome. Thiamine is considered essential for bacteria [80], and thus, the TBP can only be lost if enough thiamine is available in the environment. Fruit, the main food substrate of *Drosophila* under natural conditions [68] and the basis for the food used in our study, is mostly poor in thiamine [81]. However, other bacteria that are associated with *Drosophila*, for example other strains of *Gluconobacter* (this study), *Acetobacter pomorum* or *Lactobacillus plantarum*, can produce thiamine [37, 39]. Under these conditions, it might be beneficial for a community member to lose TBP as a result of selection for reduced metabolic expenditure [82]. This is consistent with TBP-dependent fitness effects on the host being a byproduct of selection on thiamine production in the microbe.

Our study suggests that HGT to host-associated microbes could quickly increase host fitness. An increase in microbe-mediated host fitness should also increase selection pressure on the host to favor that particular microbe that provides an increased benefit [41, 83, 84]. Waterworth et al. [85] suggested that the acquisition of genes to produce a defensive compound via HGT was key to the domestication of a bacterial defensive symbiont in beetles. We speculate that similar scenarios might be plausible for nutritional benefits in *Drosophila* because (i) mechanisms of host selection work efficiently for environmentally acquired bacteria [86–89]; (ii) stable, strain-specific associations of *Drosophila* with mutualistic bacteria have been reported [50]; and (iii) evidence for host selection in the fly is accumulating in the laboratory [45, 90] as well as under natural conditions [55, 57].

## Conclusion

Because the result of HGT here provides a potential benefit to the host under thiamine poor conditions that are often encountered under natural conditions, e.g., on thiamine poor fruit, our study contributes to a broader view of adaptation that can involve a flexible microbiome [4, 91].

## Methods

### Fitness assays

Canton-S stocks were kept at 25 °C on a 12:12 light:dark cycle on food prepared following the Bloomington *Drosophila* Stock Center “Cornmeal Molasses and Yeast Medium” (532-ml water, 40-ml molasses, 6.6-g yeast. 32.6-g cornmeal, 3.2-g agar, 2.2-ml propionic acid, and 7.6-ml Tegosept). To generate axenic flies, embryos were collected and washed in PBS, dechorionated in 50% bleach for 2–3 min, and rinsed in sterile PBS for 1 min. Embryos were placed in sterilized food bottles under a sterile workbench and maintained at 25 °C under a 12:12 light:dark cycle in axenic condition for 3 weeks during which the flies had time to hatch and mate. One axenic female from these bottles was used per vial in the fitness assay. For the fitness assay, bacterial cultures were grown in liquid YPD medium for 48–72 h and normalized to the same optical density (OD600 = 0.6). One hundred fifty microliters of OD normalized medium was added directly on 10-ml sterile grape juice food (667-ml water, 333-ml Jacoby white grape juice, 8-g yeast, 50-g cornmeal, 10-g agar, 3-ml propionic acid). The food was autoclaved without proprionic acid and proprionic acid added after the food had cooled down. Please note that yeast can in principle serve as a thiamine source, but autoclaving might have reduced the thiamine content of the food, as thiamine is heat labile [92, 93], such that it became a limiting factor for offspring number. Axenic females were transferred to the vial immediately after addition of the bacterial culture. We prepared two control treatments. First, we added sterile YPD medium to the food as axenic control. Second, we used conventionally reared flies homogenized in YPD as inoculum. On the day 16, flies were counted, collected, and weighed. All offspring were weighed together in one Eppendorf tube for each replicate and weight per fly was calculated. All fitness-related measurements were done blind. That means the vials were given random numbers and only after the measurements were taken, the bacterial strain ID was connected to the result. For the thiamine supplementation experiment, food was prepared as described above but we added 1 μg/ml thiamine to the food after autoclaving. That concentration has proven effective for phenotypic rescue in [37]. All statistical analyses were performed in R and can be found in Additional file 4: script S1.

### Bacterial loads and contamination control

Fly offspring from the fitness assays were stored in PBS/glycerol mixture at −80 °C for later contamination control and the counting of colony forming units (CFUs). Please note that glycerol is a standard cryoprotectant that allows to keep bacteria alive at −80 °C for extended time periods [94]. Effective conservation of live bacteria with this method is supported by CFU counts in the range of 10^2^–10^5^ CFUs (Additional file 6: Table S4) per fly for the majority of our samples, matching expectations from the literature well [67]. Finally, all samples underwent the freezing procedure in our randomization scheme that should prevent systematic treatment effects. Nonetheless, we cannot fully exclude that strain variation in the response to cryopreservation might have affected colony counts. For CFU enumeration 3–6 replicates per bacterial isolate were picked. To this end, samples of 3 to 5 offspring were homogenized with a pestle in 300 μl of PBS. The homogenates were plated on YPD agar medium. Plates were incubated for 48 h. CFU counts were done visually or with the OpenCFU software [95] (Additional file 6: Table S4). Plates for CFU counting were also inspected for colony morphology and colony color that could indicate potential contamination, with negative results. All homogenates were plated on antibiotic YPD agar medium (with 100 μg/ml kanamycin or ampicillin) for assessing yeast contamination. No yeast colonies were observed except in the control replicates in which conventional lab microbiota were used. To further assess potential bacterial contaminants during our experiment, we quantified the relative abundance of target isolates that flies were inoculated with on fly offspring using 16S rRNA gene sequencing (Additional file 2: Figure S10). In short, DNA was extracted from pools of 3–5 offspring for 3–6 replicates per bacterial isolate after the experiment, including the replicates with the highest and lowest offspring number. The V4 regions of the bacterial 16S rRNA gene were amplified and sequenced on an illumina MiSeq sequencer following [56, 96]. Sequencing data were analyzed using mothur [97] (see Additional file 7: script S2 for all commands executed). The relative abundance of target 16S rRNA gene sequences for mono-associated isolates was calculated. The average relative abundance of target 16S sequences was over 88% (Additional file 2: Figure S10A) in the initial experiment. Only in (6 out of 66) replicates the relative abundance was below 75%, including 3 cases of *P. sneebia* that showed very low bacterial loads. For the thiamine treatment, the target bacteria were significantly enriched in the microbial community, but there was also some evidence for contaminating 16S gene sequences, which were likely introduced during the PCR or sequencing steps (Additional file 2: Figure S10B).

### Bacterial isolates, genome sequencing, and assembly

We sequenced, assembled, and annotated draft genomes of eleven bacteria and added genome data for six bacteria from public databases (Additional file 1: Table S1). Nine strains were isolated from wild-caught *Drosophila* collected in the San Francisco Bay Area (California, USA). Isolates were cultured in YPD for standard phenol-chloroform DNA extraction. Bacterial genomes were sequenced using Illumina MiSeq technology and assembled with the A5 MiSeq assembler [98]. Completeness and contamination were assessed with checkM v1.1.2 [99], using standard settings. Assembly statistics were generated with QUAST v5.0.2 [100]. Annotation was performed with prokka v1.1 [101] or imported from GenBank. Average nucleotide identity (ANI) was computed with fastANI (v0.1.2). New isolates were taxonomically classified, using GTDBtk (v0.1.4) [102]. FastANI and GTDBtk were run on the kbase web interface [103].

### Pan-genome clustering and phylogenetic trees

Genomes were analyzed using the panX analysis pipeline [60] with standard parameters (Additional file 8: script S3). Genes were grouped into 11,269 clusters of homologous sequences, including clusters with a single gene. Thereby, the presence and absence of each gene cluster in the 17 genomes was estimated. Based on the alignments of all 134 inferred single-copy gene clusters that are present in all 17 genomes, panX reconstructs a phylogenetic tree (Fig. 1). For this phylogeny, FastTree 2 [104] and RaxML [105] were applied to all variable positions from these alignments. To create bootstrap values, we used a separate raxml call with the -b option based on the alignments created by panX (see Additional file 8: script S3). For the phylogeny of gene clusters, nucleotide sequences were aligned using MUSCLE v3.8.425 [106] and the tree was built by MrBayes 3.2.6 [62], using a molecular clock with default parameters in the Geneious software suit v1.1 (Biomatters ltd.).

### Microbial pan-genome-wide association study

We calculated the gene presence absence association score (PA score) between each predicted cluster of homologous genes and fly offspring number. That is, if *D*_g_ is the difference between the mean fly offspring for strains with and without gene g, *σ* is the global standard deviation of fly offspring for all strains and *n*_g_ is the number gene gains and losses as inferred from the phylogeny. The association score is given by$\sqrt{n_{\mathrm{g}}}\frac{D_{\mathrm{g}}}{\sigma }$. Three alternative association scores from treeWAS [21] and the corresponding model-based *p*-values were calculated. Association scores based on the presence and absence of genes are prone to false positives because genome wide linkage results in strongly correlated presence and absence of genes. PanX and treeWAS reduce this effect by taking the reconstructed ancestral gene gain and loss events into account.

## Supplementary Information

**Additional file 1. Table S1.** List of bacterial strains used in the experiments including assembly information.

**Additional file 2: Figures S1–S10. Fig.S1.** Distribution of PA scores. **Fig. S2.** Offspring weight. **Fig. S3.** ThiC and thiD missing at syntenic loci. **Fig. S4.** Synteny in Swingsia samuiensis and Neokomagateaa tanensis for thiC, thiD, and thiOSG. **Fig. S5.** Detailed view of thiamine operon insertion locus with flanking genes. **Fig S6.** Phylogeny of the HGT TBP genes thiC, thiD, and thiOSG. **Fig. S7.** Thiamine operon loci in potential donors. **Fig. S8.** GC-content at putative thiamine operon insertion sites. **Fig. S9.** multiple sequence alignment of 16S rRNA genes. **Fig. S10.** Contamination control using 16S rRNA gene sequencing after the experiment.

**Additional file 3: Table S2.** Full Panx PA scores and Treewas results table.

**Additional file 4: Script S1.** statistical analyses.

**Additional file 5: Table S3.** Blast results for HGT operon.

**Additional file 6: Table S4.** Fitness experiment data (offspring number, CFUs, fly weight).

**Additional file 7: Script S2.** 16S rRNA gene sequence analysis with mothur.

**Additional file 8: Script S3.** Microbial GWAS.

## Data Availability

The sequencing data generated and analyzed during the current study are available in the NCBI SRA repository, https://www.ncbi.nlm.nih.gov/bioproject/PRJNA656529 [107]. The bacterial genomes and the assemblies are either available under SRA number SRS7200184 – SRS7200194 [107] or from the sources described in Additional file 1: Table S1 [108–113] with raw data available in [114–119]. The 16S rRNA gene sequences are available under SRA number SRS7426971 - SRS7427068 [107] with the sample titles corresponding to the column “name_in_mothur” in Additional file 6_Table S4. Sequences of the closely related species used for the alignment in Additional file 2: Figure S4 are from [120, 121] with raw data available in [122, 123].
