# Supplementary material for: Horizontal gene transfer-mediated bacterial strain variation affects host fitness in Drosophila
Source: BMC Biol. 2021 Sep 27;19:187. doi: 10.1186/s12915-021-01124-y (PMC8474910; doi:10.1186/s12915-021-01124-y)
Supplement: Supplementary file 2 — Additional file 2: Figures S1–S10. Fig.S1. Distribution of PA scores. Fig. S2. Offspring weight. Fig. S3. ThiC and thiD missing at syntenic loci. Fig. S4. Synteny in Swingsia samuiensis and Neokomagateaa tanensis for thiC, thiD, and thiOSG. Fig. S5. Detailed view of thiamine operon insertion locus with flanking genes. Fig S6. Phylogeny of the HGT TBP genes thiC, thiD, and thiOSG. Fig. S7. Thiamine operon loci in potential donors. Fig. S8. GC-content at putative thiamine operon insertion sites. Fig. S9. multiple sequence alignment of 16S rRNA genes. Fig. S10. Contamination control using 16S rRNA gene sequencing after the experiment. [file 12915_2021_1124_MOESM2_ESM.docx]

Overview:

Figure S1 distribution of PA scores

Figure S2 offspring weight

Figure S3 thiC and thiD missing at syntenic loci

Figure S4 synteny in *Swingsia samuiensis* and *Neokomagateaa tanensis* for thiC, thiD, and thiOSG

Figure S5 detailed view of thiamine operon insertion locus with flanking genes

Figure S6 phylogeny of the HGT TBP genes thiC, thiD, and thiOSG

Figure S7 thiamine operon loci in potential donors

Figure S8 GC-content at putative thiamine operon insertion sites

Figure S9 multiple sequence alignment of 16S rRNA genes

Figure S10 contamination control using 16S rRNA gene sequencing after the experiment

Table S1 list of bacterial strains used in the experiments including assembly information

Table S2 full PA and Treewas results table

Table S3 blast results for HGT operon

Table S4 fitness experiment data (offspring number, CFUs, fly weight)

Script S1 statistical analyses

Script S2 16S rRNA gene sequence analysis with mothur

Script S3 microbial GWAS


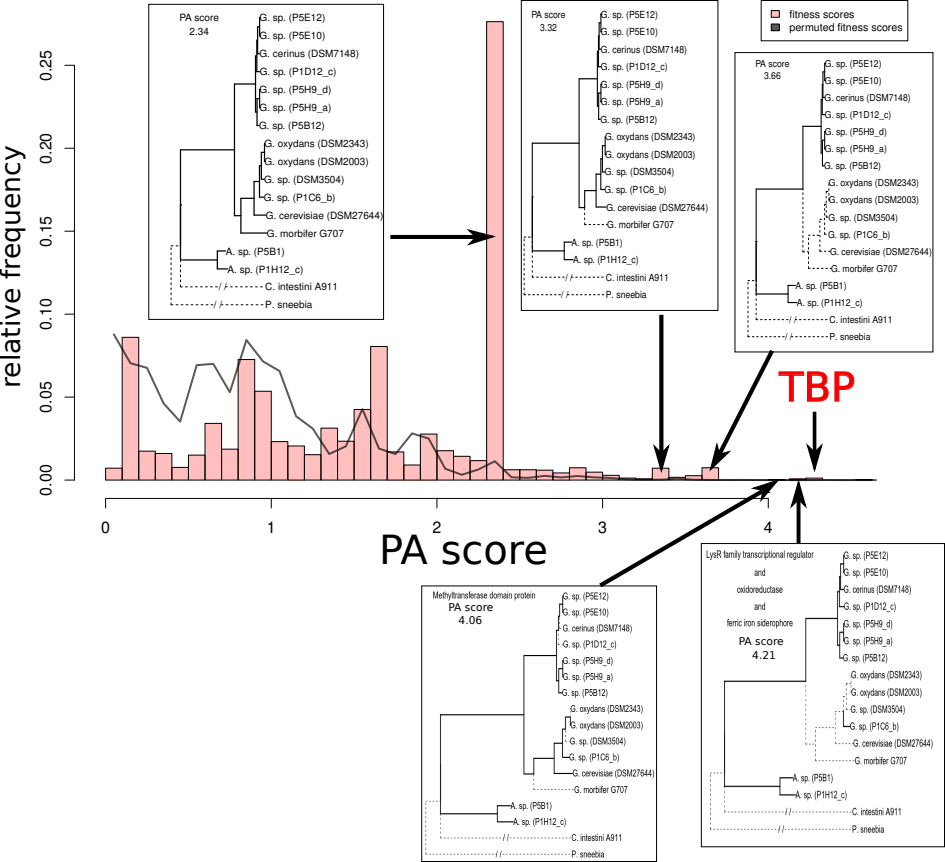
**Figure S1** Distribution of PA-scores. The presence absence score (PA score from PanX [60]) for all *Gluconobacter* clusters of homologous genes is shown as a bar chart. The TBP genes have a score of 4.34. The peak at 2.34 corresponds to the genes that are missing in the outgroup strains *C. intestini* and *P. sneebia*, but present in all other strains (phylogeny to the left). The peak at 3.32 corresponds to the genes that are missing in *C. intestini, P. sneebia,* and *G. morbifer* G707. The peak at 3.66 represents the genes that are private to the *G. cerinus* clade and shared with *Acetobacter*. Only with strain variation within the *G. morbifer*/G*. oxydans* clade as shown in Figure 1, we get the extreme PA scores for the TBP. The solid line represents the PA score distribution for 10 datasets with randomly permuted fitness values. We found one cluster with an association score higher than 4.34 in the permuted data sets. This corresponds to the PA scores for the TBP genes being in the 99.999 percentile. The mean PA score for TBP genes from permutations is 0.86 +/- 0.59. Presence-absence patterns for the other candidate genes from Table 1 are shown below the graph. Box to the right: LysR family transcriptional regulator, oxidoreductase, and ferric iron siderophore receptor at PA score 4.21; box to the left: methyltransferase domain protein at a PA score of 4.06.


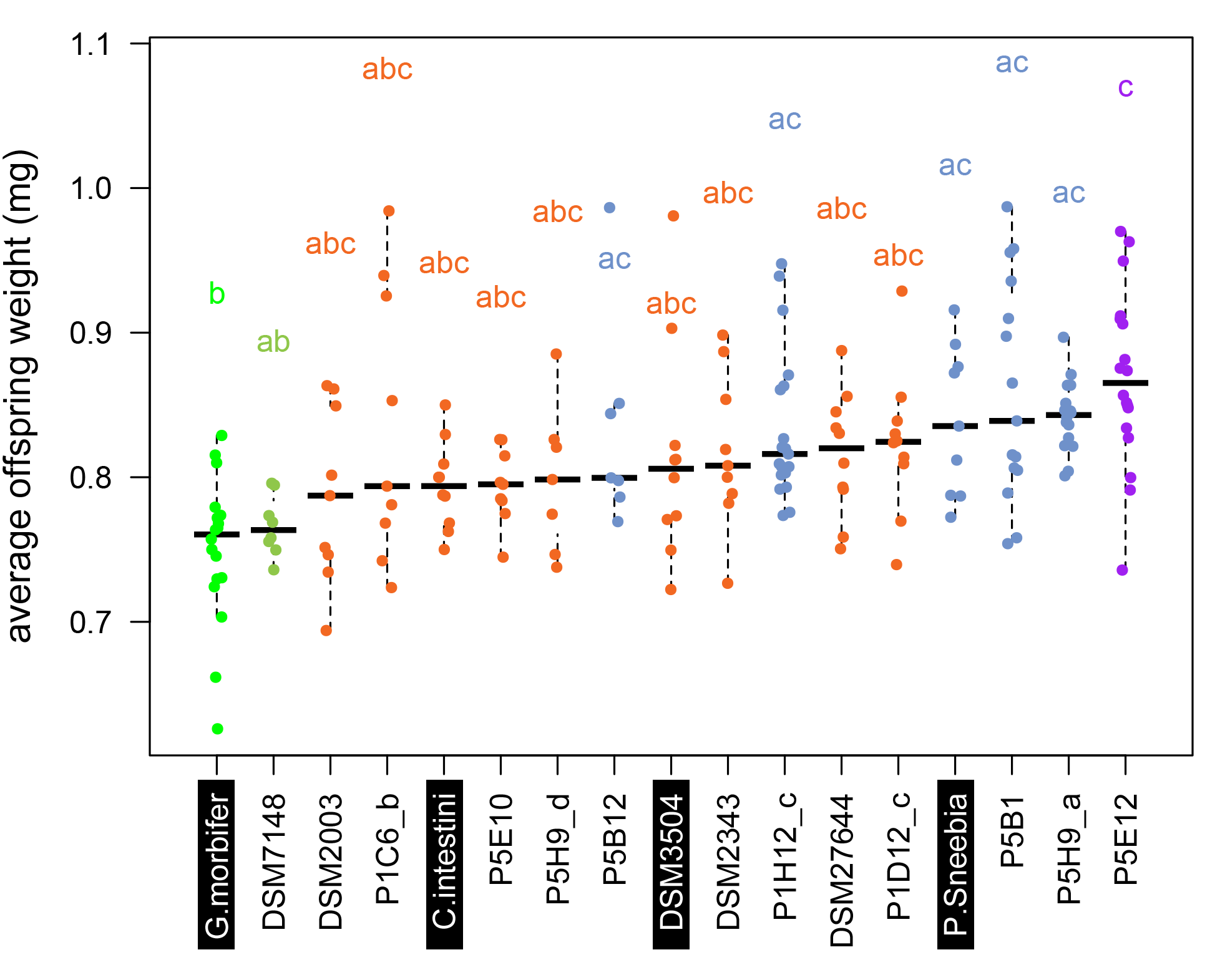
**Figure S2** Weight per offspring from mono-associated female flies. Offspring weight did not differ significantly between TBP+ and TBP- strains. Isolates that do not share one of the letters above (a,b,c) differ significantly according to Tukey’s test.


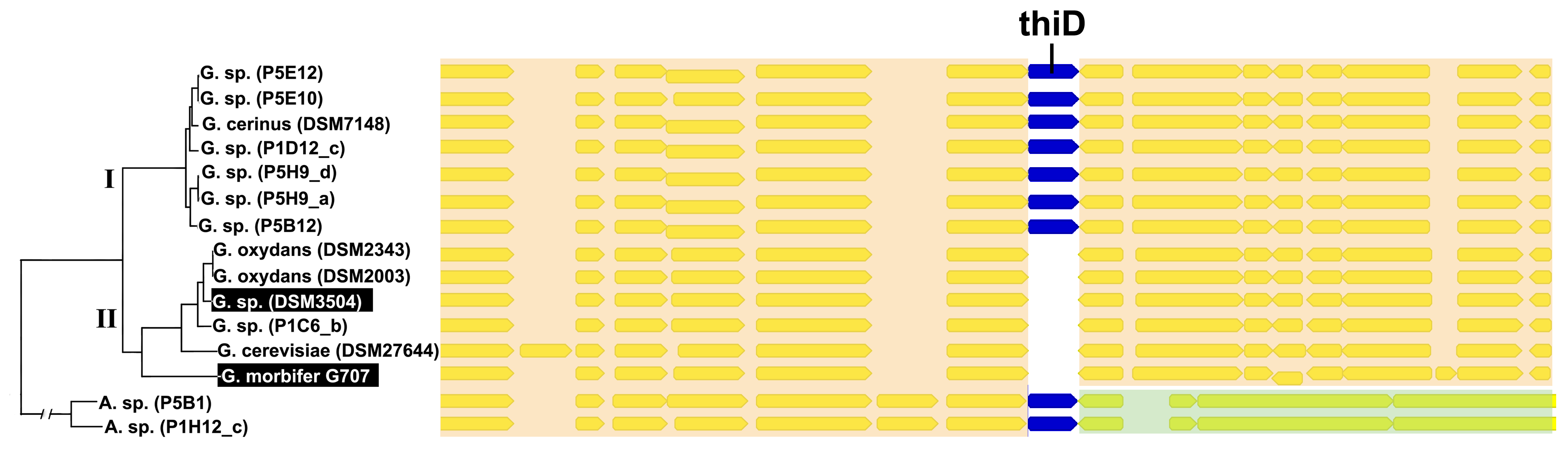


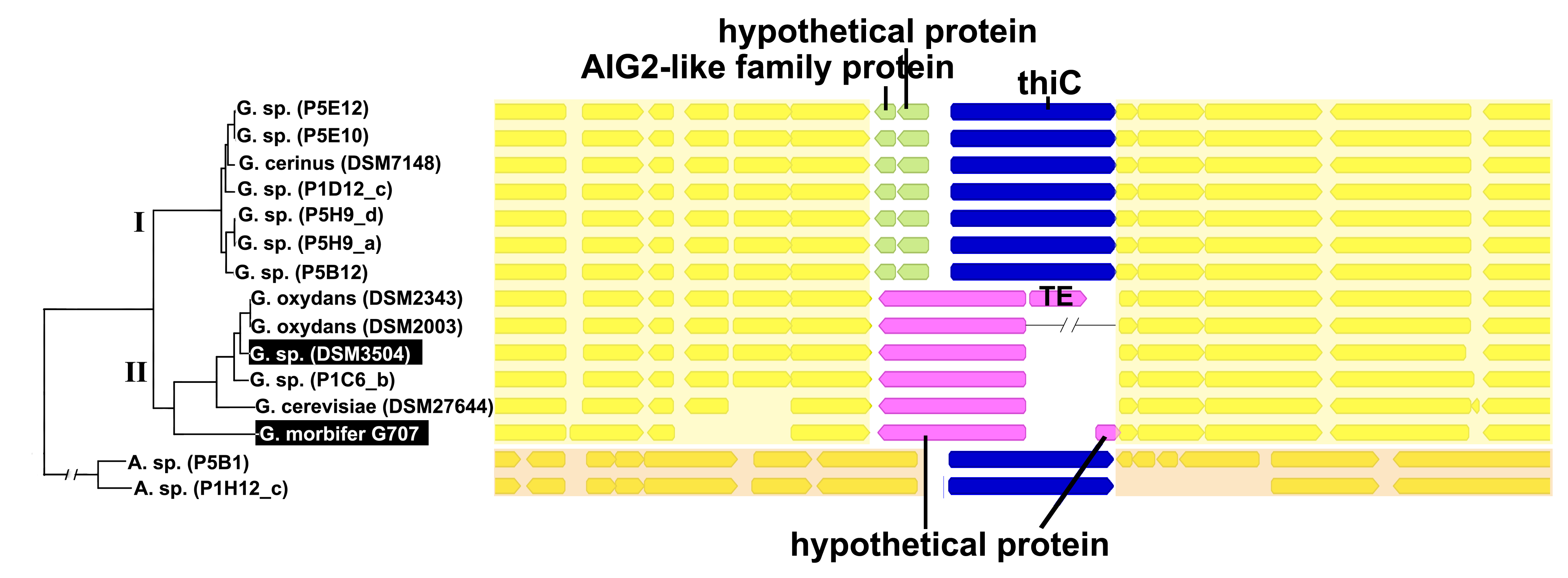
**Figure S3** thiC and thiD are missing at syntenic loci on the *G. morbifer* branch.


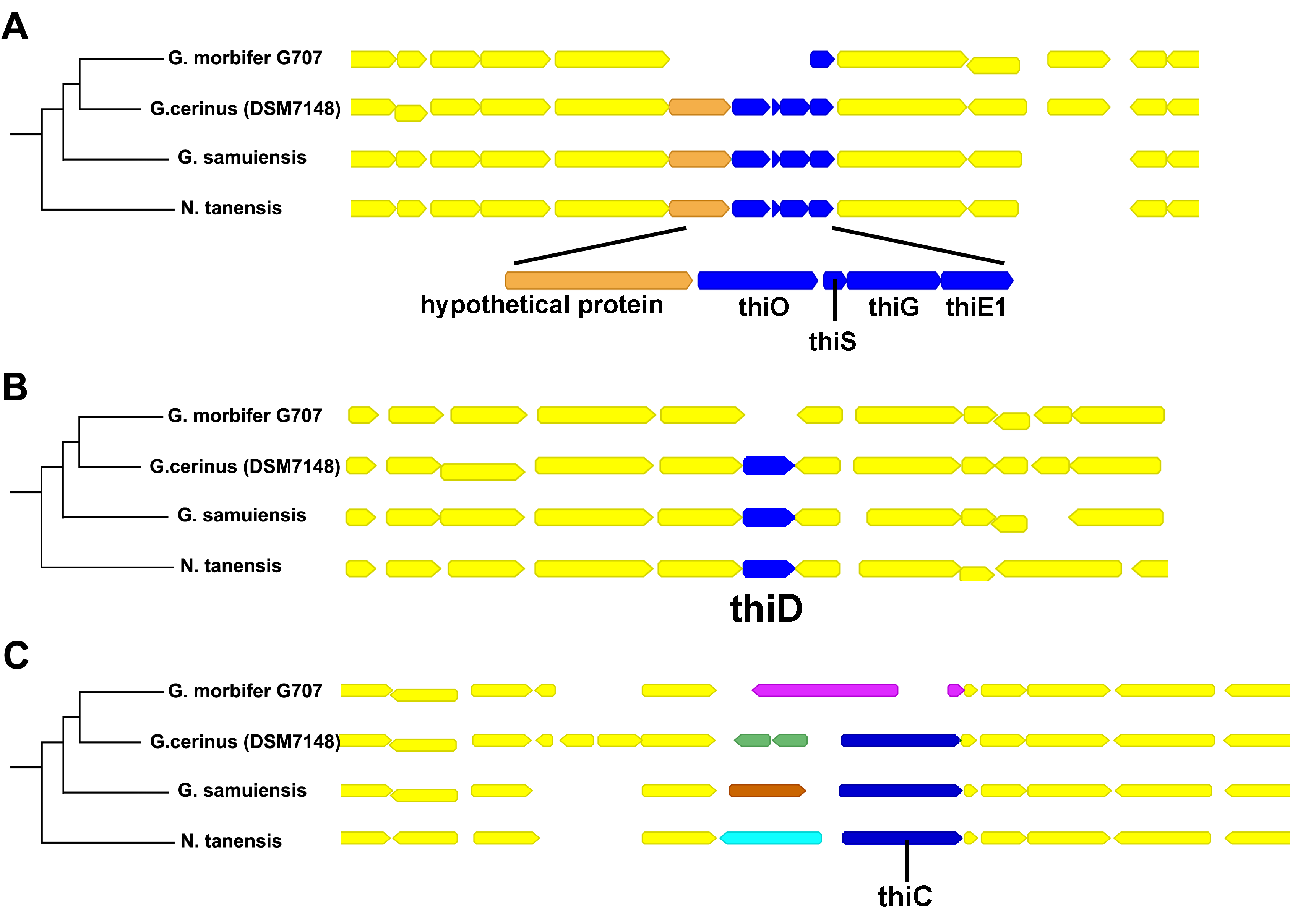
**Figure S4** The closely related species *G. samuiensis* (also called *Swingsia samuiensis*, NCBI Reference Sequence: NZ_CP038141) [122] and *Neokomagateaa tanensis* (NCBI Reference Sequence: NZ_CP032485) [123] possess thiOSG at the syntenic locus pointing towards a deletion on the *G. morbifer* branch. The phylogeny is based on GTDB v95 [102]. We find the same pattern for thiC and thiD.


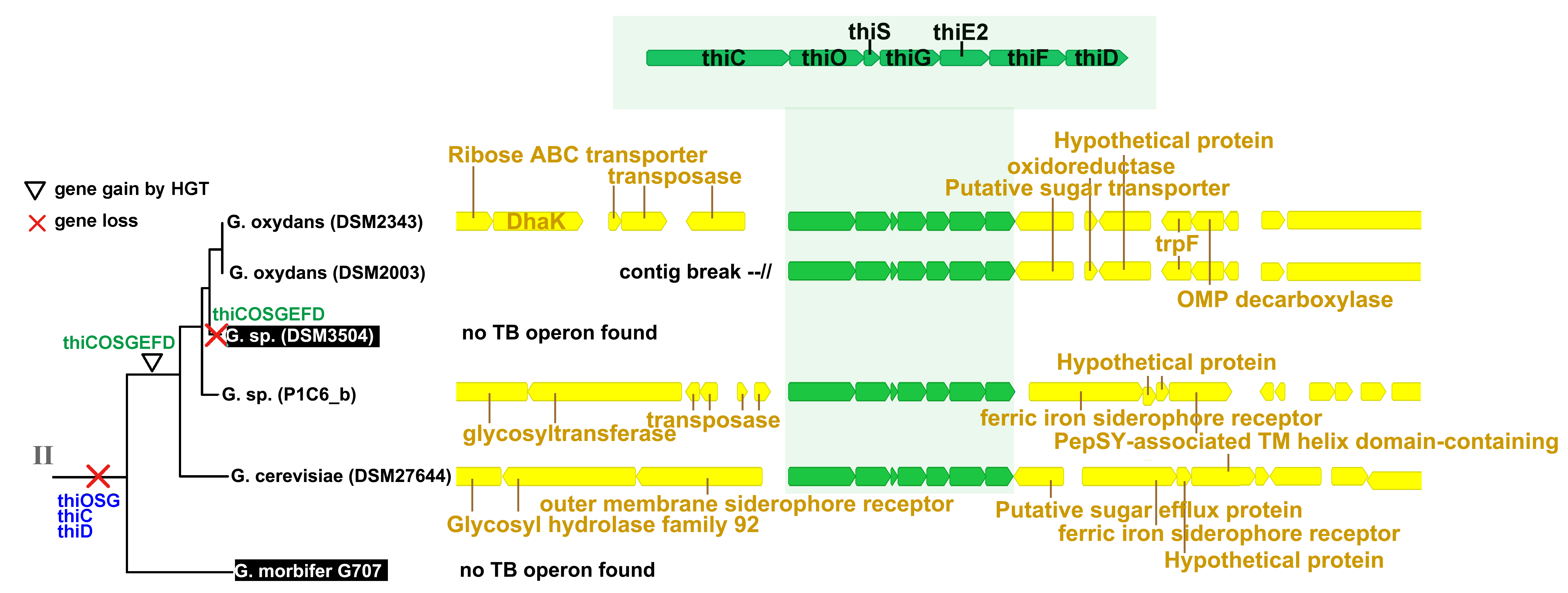
**Figure S5** Detailed view of thiamine operon insertion locus with flanking genes. The putatively inserted thiamine operon on branch II that is shown here is in a different locus than the locus that was deleted in branch I (Figure 2C) as evident from the flanking genes. Also, within branch II, the thiamine operon can be found at different loci, as is also evident from the flanking genes. Despite the different locations of the operon in the genome, a single acquisition event seems most plausible because the phylogeny of the putatively HGT thiamine operon follows the core gene phylogeny (Figure S6). Transposases located upstream of the operon might have been involved in reshuffling the location of the operon after insertion.

**
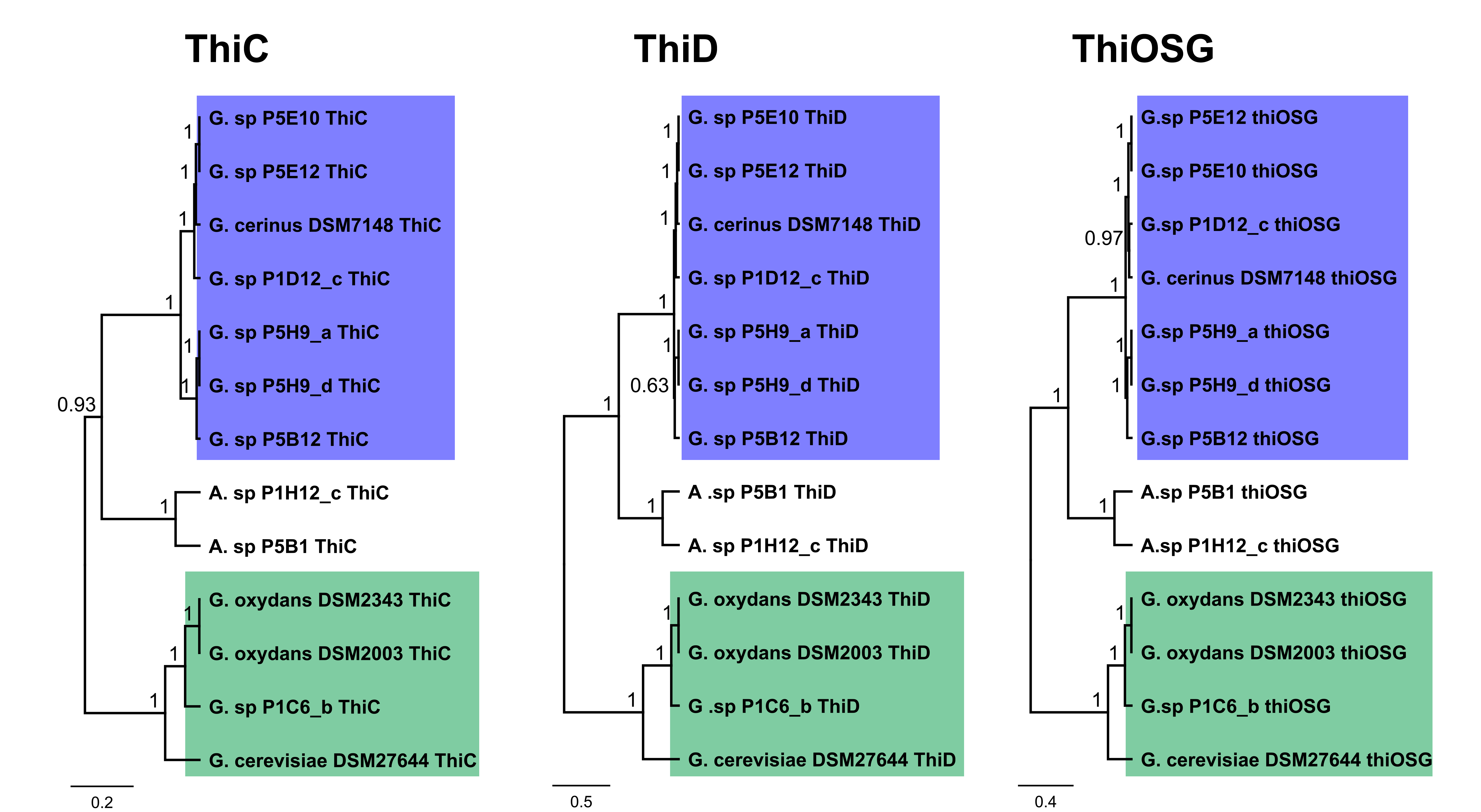
**

**Figure S6** The phylogeny of the TBP genes (thiC, thiD, thiOSG) is incongruent with the core phylogeny. The TBP genes that were acquired by HGT in the strains *G. oxydans* DSM2343, *G. oxydans* DSM2003, *G. sp.* P1C6_b, and *G. cerevisiae* DSM27644, form a distant clade (green background). Within this clade the phylogeny is congruent with the core phylogeny further supporting a single HGT of the operon to the *G. morbifer* branch (branch II in Figure 2). The nucleotide sequences of thiC, thiD and thiOSG were aligned using MUSCLE v3.8.425 [106] and the tree was built using MrBayes 3.2.6 [62] as incorporated in Geneious Primer 2020.1.1 (Biomatters ltd.). Node labels represent posterior probabilities as assessed by MrBayes.


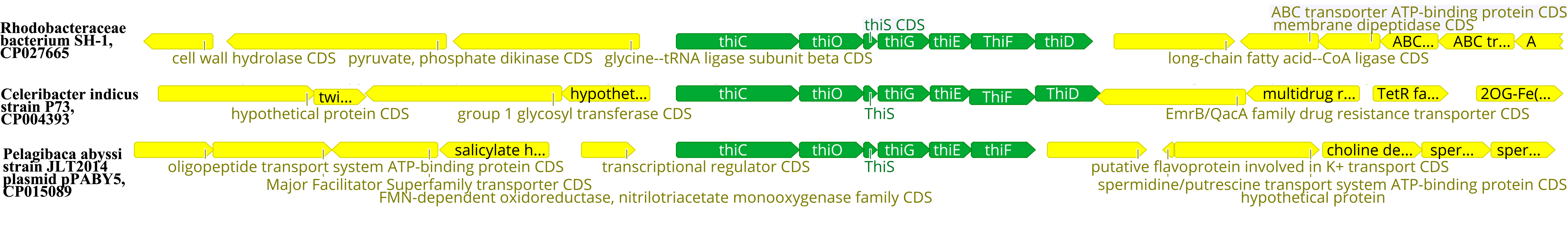
**Figure S7** Thiamine operon loci in potential donors. The order of thiamine genes is the same as for the putative HGT operon on branch II. However, the flanking genes are different.


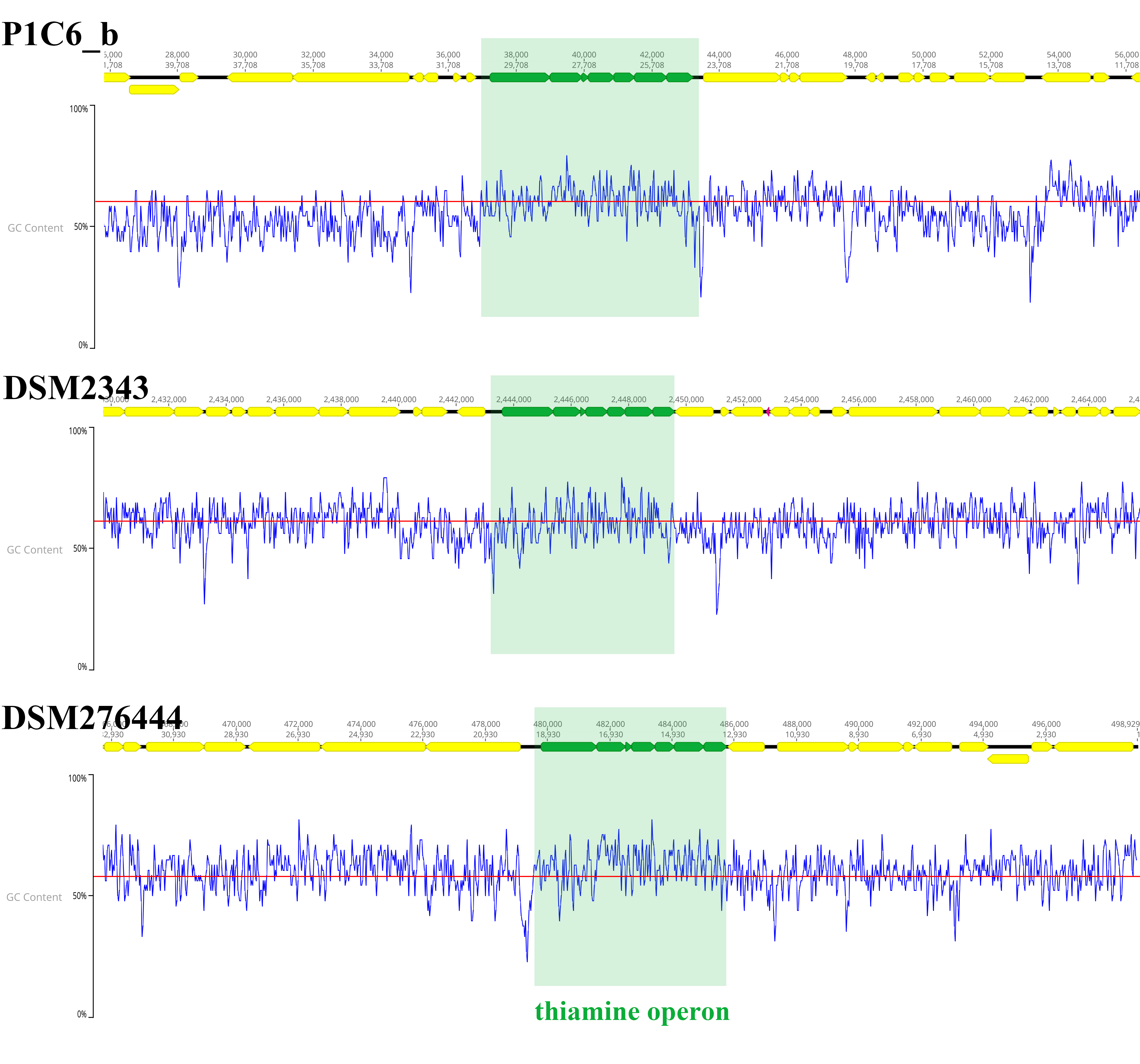
Figure S8 GC-content at putative thiamine operon insertion sites. GC-content did not markedly differ from genomic background at insertion site.


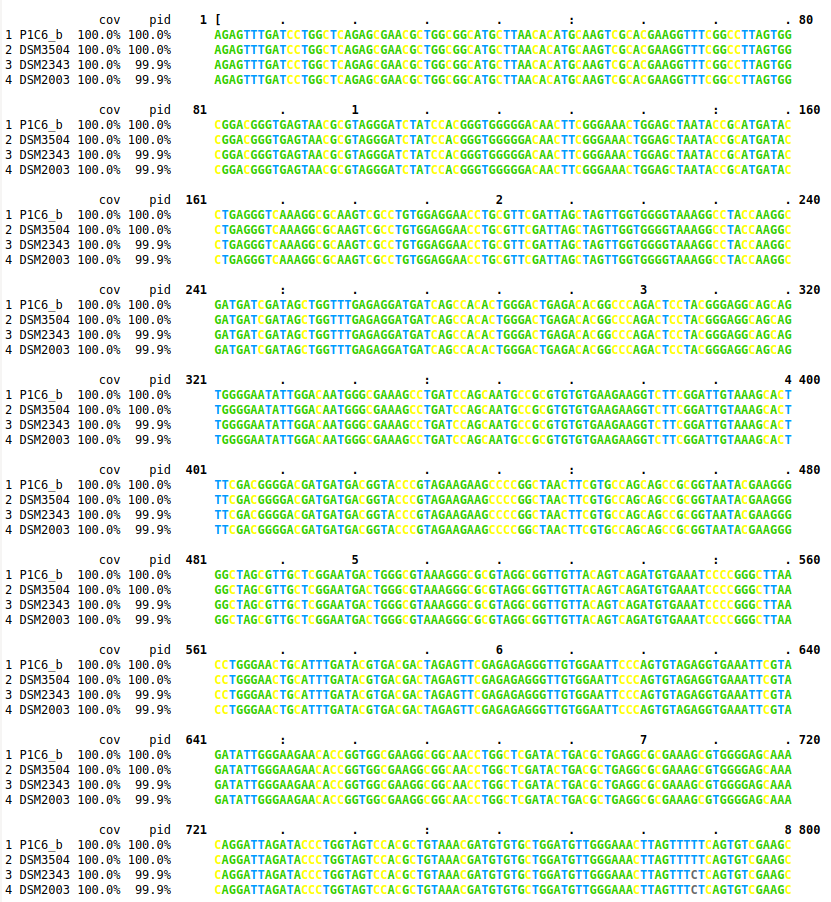


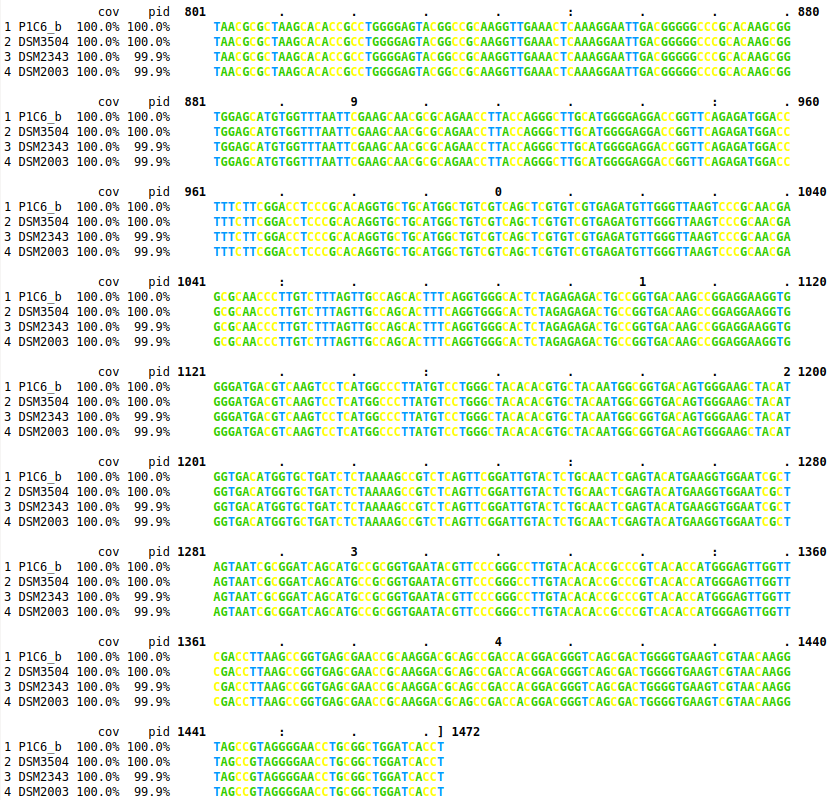
**Figure S9** 16S rRNA gene alignment of closely related *Gluconobacter* strains from branch II. Please note that 16S rRNA gene sequences from strains P1C6_b (TBP+) and DSM3504 (TBP-) are identical, thus providing an example that important strain variation with functional consequences for the host would have been missed with a 16S rRNA gene sequencing approach. Furthermore, there is only 1 bp difference at position 887 between P1C6_b and DSM3504 on the one hand and DSM2343 and DSM2003 on the other hand. This difference would have been missed by the most common 16S rRNA gene sequencing methods that target V3 or V4. The 16S rRNA gene was amplified with the primers 27F 5'-AGAGTTTGATCCTGGCTCAG-3' and 1492R 5'-GGTTACCTTGTTACGACTT-3' and sequenced using Sanger sequencing.


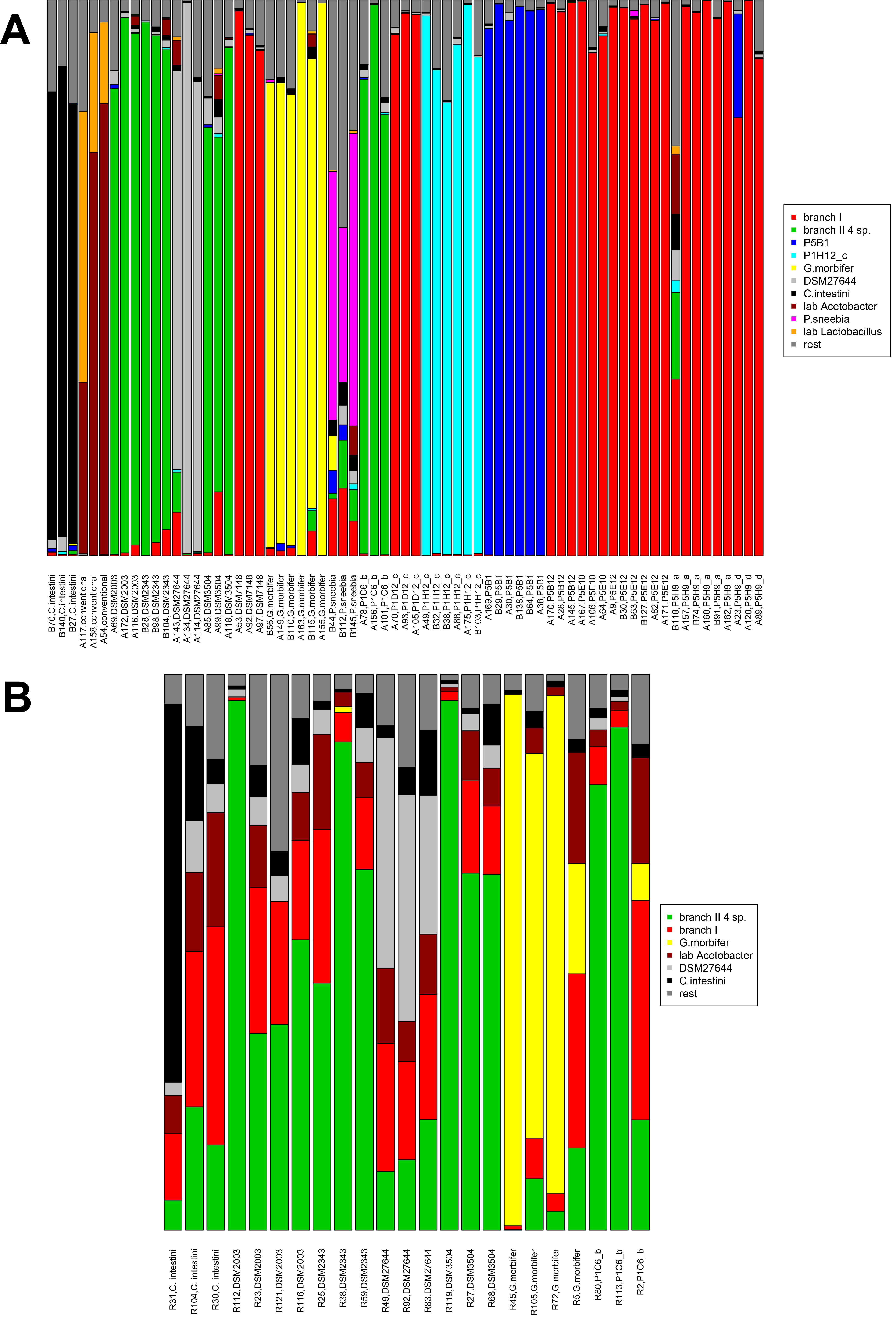


**Figure S10** The relative abundance of bacterial taxa on flies after the experiments. (A) For the initial fitness assay. The average relative abundance of target 16S sequences was over 88% in the fitness assay, indicating successful inoculation with the target bacterium. Only in 6 out of 66 replicates, the relative abundance was below 75%, including 3 cases where flies were inoculated with an isolate of *P. sneebia*. *P. sneebia* showed low bacterial loads (Table S4). Therefore it seems plausible that *P. sneebia* did not grow as well as the other strains under our experimental conditions such that low levels of contamination with 16S rRNA gene DNA had a large effect in these samples. (B) For the thiamine supplement experiment. The target isolates were significantly enriched in the replicates (P in the range of 0.000082~0.028, Mann-Whitney-Test). However, we found potential contamination (red and brown bars). Two samples (R112 and R45) showed significantly less evidence for potential contaminants. These two samples were run together with the other samples in the experiment, but underwent PCR and purification in another batch, suggesting that the contamination might have taken place during the PCR or sequencing steps and not during the experiment. Three to six replicates per bacterial strain were selected for sequencing as described in the main text. Bacterial communities were profiled by 16S rRNA gene sequencing of whole homogenized flies following Kozich et al. [96] and Wang et al. [56]. A detailed analysis script can be found as script S2 in the supplementary files.
